# An introduction to the ‘Psycho-Physiological-Stress-Test’ (PPST)—A standardized instrument for evaluating stress reactions

**DOI:** 10.1371/journal.pone.0187859

**Published:** 2017-12-01

**Authors:** Elisabeth Neureiter, Loreen Hajfani, Anne Ahnis, Annett Mierke, Matthias Rose, Gerhard Danzer, Burghard F. Klapp

**Affiliations:** 1 Center for Internal Medicine and Dermatology, Division for General Internal and Psychosomatic Medicine, Charité - Universitätsmedizin Berlin, Berlin, Germany; 2 Alpen-Adria-Universität Klagenfurt, Klagenfurt, Austria; 3 Medical University Brandenburg - Campus Ruppiner Kliniken, Division for General Internal and Psychosomatic Medicine, Neuruppin, Germany; Nagoya University, JAPAN

## Abstract

Using a standardized instrument to evaluate patients’ stress reactions has become more important in daily clinical routines. Different signs or symptoms of stress are often unilaterally explored: the physiological, psychological or social aspects of stress disorders are each viewed on a single dimension. However, all dimensions afflict patients who have persistent health problems due to chronic stress. Therefore, it is important to use a multidimensional approach to acquire data. The ‘Psycho-Physiological-Stress-Test’ (PPST) was established to achieve a comprehensive understanding of stress and was further developed at the Charité—Universitätsmedizin Berlin in collaboration with the Psychological Department of Freie Universität Berlin. The PPST includes a series of varying stress phases, embedded in two periods of rest. Physiological and psychological parameters are simultaneously measured throughout the test session. Specifically, the PPST activates the sympathetic stress axis, which is measured by heart rate, blood pressure, respiration depth and rate, electro dermal activation and muscle tension (frontalis, masseter, trapezius). Psychological data are simultaneously collected, and include performance, motivation, emotion and behavior. After conducting this diagnostic test, it is possible to identify individual stress patterns that can be discussed with the individual patient to develop and recommend (outpatient) treatment strategies. This paper introduces the PPST as a standardized way to evaluate stress reactions by presenting the results from a sample of psychosomatic inpatients (n = 139) who were treated in Charité—Universitätsmedizin Berlin, Germany. We observed that the varying testing conditions provoked adjusted changes in the different physiological parameters and psychological levels.

## Introduction

Given the problems with the psychophysiological discipline’s multidisciplinary approach, we present an economical stress test for evaluating stress reactions in the clinical routine. Patients who suffer from body related complaints, in the context of psychosocial distress, are often referred to a psychosomatic expert after several years. Prior to the psychosomatic consultation, these patients have usually completed a large number of physicals without pathological results [1,2]. It is often difficult for patients to understand the relation between physiological and psychosocial aspects of illness. Stress is associated with a wide range of different diseases that involve almost every physiological system, such as cardiovascular, gastrointestinal and respiratory system [3–5]. Many clinical studies have experimentally verified that stress affects individual’s physiological and psychological systems [6–9]. Selye defined stress as the body’s nonspecific reaction to any demand [10]. In 1975, Selye [11] differentiated between ‘dis- and eustress’, or pathological stress (negative, distress) vs. health-promoting stress (positive, eustress). Although distress leads to (severe) physiological and psychological health problems, eustress has beneficial outcomes, including the ability to adjust to new situations or focus on solving problems, for example, at work [12]. Lazarus and Folkman postulated, that stress is a pattern of a negative psychophysiological condition in which individuals are (or feel) unable to cope with situations which they perceive as threats to their well- being [13]. Stress and distress evoke person-, organ- and stimuli-specific (stress) reactions. Therefore, it is important to assess a wide range of physiological and psychosocial parameters when studying individuals’ stress reaction(s).

Other multidimensional stress tests that are comparable with the PPST are the ‘Trier Social Stress Test (TSST)’ [14], the ‘Trier Mental Challenge Test’ (TMCT) [15] and the ‘Mannheimer Multikomponenten Stress Test’ (MMST) [16]. These psychophysiological instruments often examine physiological parameters, such as salivary cortisol, heart rate and heart rate variability [17].

In our experimental setting, patients are exposed to stimuli in varying conditions that are supposed to evoke psychophysiological activity. As such, we perform a statistical analysis with 139 unselected inpatients with the usual spectrum of diseases of a psychosomatic division (i.e., adaptive disorders, somatoform disorders, affective disorders, stress related disorders) to test the hypothesis that the PPST detects stress reactions in the biopsychosocial dimensions of interest.

## Methods

### PPST protocol

**Fig 1:**
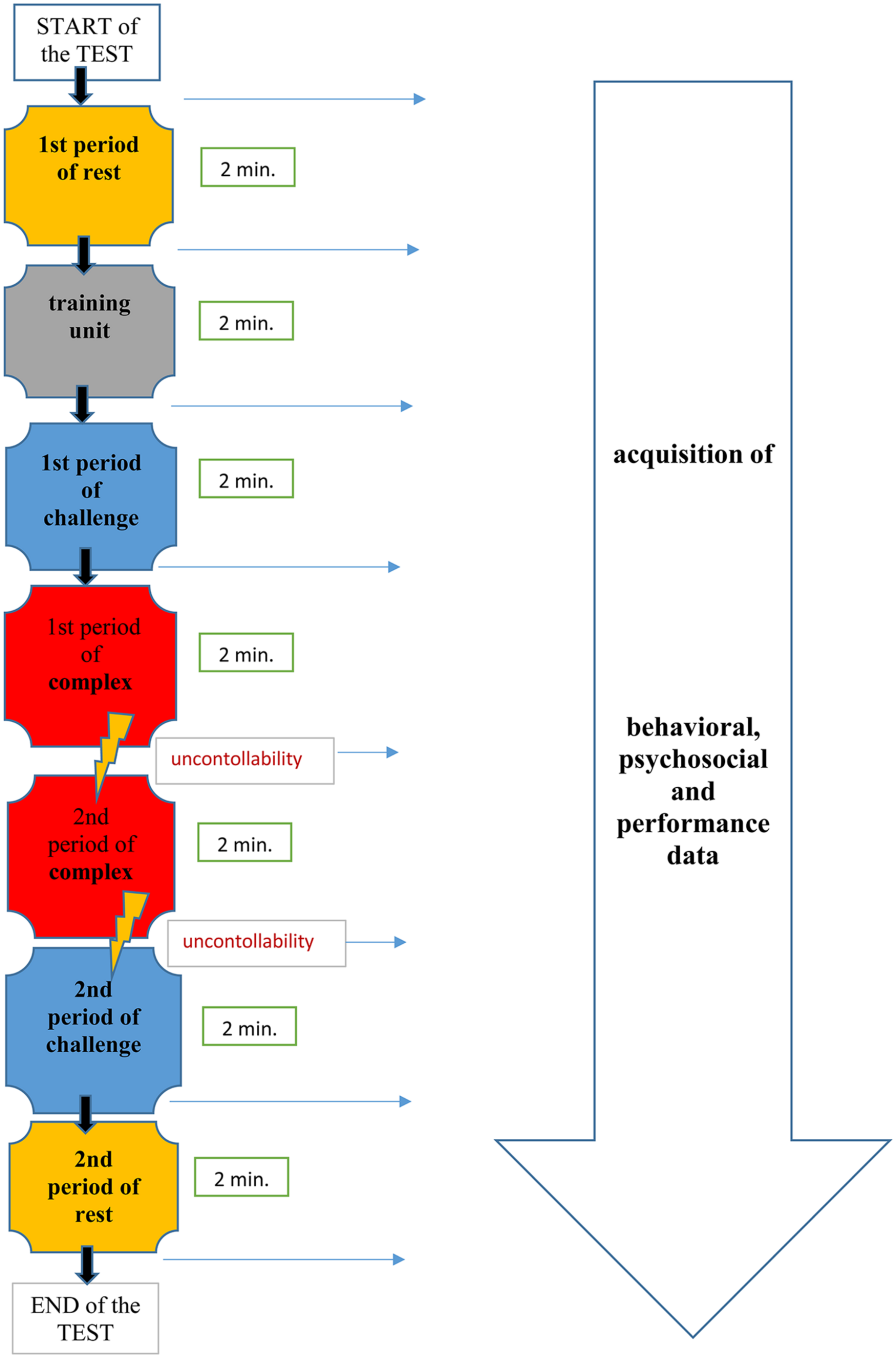
PPST protocol.

The PPST consists of periods of (simple) challenge and complex tasks that should result in stress or distress and two periods of rest. Each period lasts for approximately 2 minutes (Fig 1). Between the testing periods, the patient is provided with additional instructions for the next tasks, which includes providing psychological data to measure emotions, motivations, expectations and performance.

The patient’s task is to find two numbers from a random matrix of 36 numbers (in the range of 01 to 99) as quickly as possible. The patient has four options to report their presence: number one or number two is present, both are present or neither is present.

The test begins with a first rest period that is indicated by a standardized picture on the monitor and serves as a baseline for the physiological variables. This period is followed by a training unit. The training unit familiarizes the patient with the task. Then, the main test is initiated, and the first period is a challenge in which the patient must perform the task as quickly as possible; the matrix presentation depends on the patient’s pace of responding. For correct and incorrect answers, patients receive visual feedback at the bottom of the computer screen. A green smiley face is shown for correct answers. For incorrect answers, a red glum emoticon arises, and the patient receives an acoustic signal. The next two periods are complex task periods that are characterized by the computer’s algorithm, which is a matrix presentation that accelerates based on how quickly the patient responds, resulting in stress or distress: The patient has no influence on the task’s timeframe. The computer calculates an algorithm that sets the pace and is based on achieving the maximum 40% correct answers. The participant is instructed that his peer group, on average, responds correctly 60% in the complex task periods. This discrepancy is an additional stress component (a social reference in addition to a loss of control for performance to create a defining criterion of distress). After these two forced task periods, there is an additional period in which the patient proceeds at his own pace and a second rest period prior to the test completion.

The experimental session lasted approximately 40 minutes (minimum 30 min. & maximum 60 min). After arriving at the laboratory, patients were provided with a standardized introduction about the test procedure and the computer simulation. Immediately prior to beginning the stress test, the examiner, who was a psychologist, checked the placement of the line cables that were used for the physiological parameters (heart rate, blood pressure, respiration, electro dermal activation and the muscle tension—see the following paragraph). After the test and removing the test equipment, patients are provided with detailed analyses about their PPST results with cautious interpretations by the examiner. Together, the patient and examiner identify patterns that resemble coping strategies or physiological stress reactions and develop and discuss possible ambulatory treatment options.

### Physiological parameters

The PPST software runs on a standard desktop computer and presents all instructions, questions, visual analogue scales and visual search tasks to the patient while controlling data acquisition via a serial connection (RS232) to the data-acquisition computer. The data are stored as ASCII files and are processed offline with Excel 2007 to produce the test report. The ECG (Electrocardiography) was recorded with a modified Einthoven II Lead that used Kendall H34SG electrodes. The EMG (Electromyography) was recorded as bipolar with Kendall H124SG electrodes. EDA (Electrodermal activity) was recorded thenar and hypothenar (the palm of left hand) with Kendall H34SD electrodes. Respiration was recorded with a respiration belt. These signals were measured by a Nexus10 device (MindMedia Inc., NL) that sampled at 1024 Hz and transmitted to the data-acquisition computer via bluetooth. Blood pressure and pulse were continuously measured from the left middle finger with an Ohmeda 2300 Finapres device. Every 2 minutes, the arterial blood pressure from the right arm was auscultatorily measured with a Dinamap 1846sx. Both blood pressure measuring devices send their data to the data-acquisition computer via a serial connection.

The recording software was programmed with DasyLab 8.0.4. The ECG was high-pass (1 Hz) filtered and processed to RR-intervals to compute the heart rate and RMSSD (root mean square of successive differences, HRV—Heart Rate Variance). The three EMG signals were high-pass (20 Hz) filtered and processed with a root mean square (RMS) algorithm that had a time constant of 125 ms. EDA was recorded as skin conductance level (SCL) and was high-pass (0.5 Hz) filtered to obtain the skin conductance response (SCR). The respiration signal was high-pass (0.6 Hz) filtered, and the breathing depth and rate were computed online. Artefacts were automatically removed by a plausibility algorithm. All signals were down sampled to 4 Hz and recorded as ASCII files. These files were then processed with Excel 2007 to compute the physiological data’s means and standard deviations and to produce the graphical reports.

### Psychological parameters

Psychological data included patients’ self-reported estimations of task performance, emotions, motivations and physical discomfort. Likert scale items were used to record emotions and motivations and patients’ ideal and real expectations for the ongoing testing process. As mentioned above, these data were collected between the challenge periods. The psychological data summarizes how patients behave or cope in challenging situations (stress) that are similar to the special challenges that are presented in the PPST.

### Statistical analysis

All physiological and psychological variables were analyzed as dependent variables in a univariate ANOVA for repeated measures (within subject design—1x 7) to assess differences between the test-periods (the independent variables were the arrangements of stress test periods: rest periods 1 & 2, challenge at ones’ own pace 1 & 2; and forced task from the computer algorithm with a social reference / stress / distress 1 & 2). First, we assessed whether condition had an overall influence on the variable. Then, we analyzed contrasts to test differences between subsequent periods.

If the sphericity assumption was not met, the Greenhouse-Geisser approach was used to adjust the degrees of freedom, additionally we employed the Bonferroni adjustment for the multiple comparisons. To investigate if the medical condition of the participants has an impact to the results of the PPST, we conducted a mixed repeated-measures ANOVA with medical condition (main diagnoses) as a between—subject factor and all the test parameters as well as the test periods as within-subject factors. All analyses were conducted using SPSS Version 21.

For the physiological values that were collected during 2-min PPST periods, we computed the means per period for the SCL, SCR, three EMG signals from right sided m. trapezius, left sided m. masseter and m. frontalis, systolic and diastolic finger cuff blood pressure (SFB resp. DFB) and for heart rate (HR) and RMSSD from the ECG (HRV). Additionally, the Ohmeda 2300 Finapres was used to compute the pulse rate (PR), which was used for non-artefact-free ECG. The EDA was used to obtain the SCL and SCR means. For respiration, we used the means for each 2-min period of breathing amplitude (RA) and respiration rate (RR).

### Sample

We enrolled 139 unselected inpatients at treatment admission (i.e., internistic therapy and physiotherapy, as well as both individual and group psychotherapy, music and art therapy and body centered psychotherapy) from the Division of General Internal and Psychosomatic Medicine, Charité—Universitätsmedizin Berlin, Germany. Participants represented the average diagnostic spectrum range in our division. We found no significant differences between our study sample and all other inpatients during this study’s time period on the ‘Patient Health Questionnaire’ (PHQ) [18]; the ‘Perceived Stress Questionnaire’ (PSQ) [19]; and the COPE [20]. A descriptive summary of our sample is provided in Table 1. According to our medical doctors or clinical psychologist’s prescriptions, participating in a stress test was part of the clinical schedule for evaluating stress reactions. All patients agreed in written form to the use of their secondary data for clinical routine studies on admission to the Center for Internal Medicine and Dermatology, Division for General Internal and Psychosomatic Medicine. This study was approved by the Vote of the Charité ethics committee (EA1/114/10).

**Table 1:** Patients’ socio-demographic characteristics.

Total number of patients^a^	
	N (M)^b^
Age in years	(42,55)
Range	17–79
Gender (f/m)	93/46
^c^Employed (yes/no)	104/26
^d^Partner relationship (yes/no)	68/64
^e^**ICD-10 Diagnosis Code**	
^f^affective disorders (F30-F39)	24
neurotic, stress-related and somatoform disorders (F40-F48)	94
behavioural syndromes associated with physiological disturbances and physical factors (F50-F59)	21

^a^N = 139

^b^N = total number of patients; M = mean

^c^Employed: *N* = 9 not reported

^d^Partner relationship: *N* = 7 not reported

^e^[21]

^f^ N = main diagnoses

## Results

### Results for physiological and psychological parameters—Tables 2, 3, 4 and 5

**Table 2:** Performance parameters: Repeated measures ANOVA.

	training unit	1^st^ challenge	1^st^ complex task	2^nd^ complex task	2^nd^ challenge	p (overall)
**Total number presented; mean (SD)**	11.0 (3.7)	12.5 (3.9)	30.1 (9.4)	34.7 (12.0)	19.4 (8.7)	**< 0.001**
		**p < 0.001**	**p < 0.001**	**p < 0.001**	**p < 0.001**	
**Total number of right solutions; mean (SD)**	8.8 (3.3)	10.7 (3.5)	11.6 (4.3)	13.3 (4.9)	14.8 (4.8)	**< 0.001**
		**p < 0.001**	**p = 0.025**	**p < 0.001**	**p < 0.001**	
**right solutions %; mean (SD)**	80.2 (16.7)	86.3 (15.0)	37.7 (6.0)	37.7 (5.3)	80.0 (15.8)	**< 0.001**
		**p < 0.001**	**p < 0.001**	p = 0.996	**p < 0.001**	
**patients' estimation; mean (SD)**		77.5 (21.3)	30.3 (12.0)	29.5 (13.1)	66.4 (24.2)	**< 0.001**
			**p < 0.001**	p = 0.355	**p < 0.001**	

1^st^ and 2^nd^ challenge tasks with a self-determined speed and no comparison. 1^st^ and 2^nd^ complex tasks with a computer determined speed and a comparison with no realistically high social ‘norm’. In bold represent the significant results. The overall results of the performance parameters show F-values as listed below: Total numbers presented F(2.17, 299.58) = 459.44, p < 0.001, ƞ_p_^2^ = .77, ɛ = 1.00, Total number of right solutions F(2.71, 374.53) = 104.10, p < 0.001, ƞ_p_^2^ = .43, ɛ = 1.00, right solutions % F(2.75, 379.43) = 731.98, p < 0.001, ƞ_p_^2^ = .84, ɛ = 1.00, patients’ estimation F(2.08, 287.04) = 375.44, p < 0.001, ƞ_p_^2^ = .73, ɛ = 1.00.

**Table 3:** Physiological parameters: Repeated measures ANOVA.

	1^st^ rest	training unit	1^st^ challenge	1^st^ complex task	2^nd^ complex task	2^nd^ challenge	2^nd^ rest	p (overall)
**cardiovascular system**:								
systolic blood pressure in mmHg; mean (SD)	136.1 (23.1)	142.6 (25.4)	145.9 (26.2)	147.4 (27.2)	145.8 (27.1)	144.7 (27.7)	140.6 (26.3)	< 0.001
		p < 0.001	p < 0.001	p = 0.830	p = 0.172	p = 0.102	p < 0.001	
diastolic blood pressure in mmHg; mean (SD)	74.8 (12.0)	77.4 (12.3)	78.8 (12.9)	80.3 (13.1)	80.1 (13.4)	79.5 (13.3)	77.3 (12.8)	< 0.001
		p < 0.001	p < 0.001	p < 0.001	p = 0.496	p = 0.718	p < 0.001	
heart rate in bpm; mean (SD)	78.2 (12.8)	81.4 (13.0)	82.6 (13.2)	82.1 (12.4)	81.1 (12.2)	80.6 (12.1)	78.7 (11.7)	< 0.001
		p < 0.001	p = 0.004	p = 0.123	p < 0.001	p = 0.664	p = 0.000	
**respiratiory system**:								
breath depth in aU; mean (SD)	6.8 (7.5)	5.3 (5.1)	5.4 (5.3)	5.2 (5.1)	4.9 (4.8)	5.2 (5.1)	6.0 (6.6)	< 0.001
		p < 0.001	p = 0.226	p = 0.146	p = 0.135	p = 0.228	p = 0.199	
respiration rate in sec; mean (SD)	4.1 (1.5)	3.4 (0.6)	3.5 (1.0)	3.4 (0.7)	3.5 (0.8)	3.6 (1.0)	4.1 (1.1)	< 0.001
		p < 0.001	p = 0.349	p = 0.483	p = 0.396	p = 0.258	p < 0.001	
**muscle tension**:								
EMG_frontalis in μV; mean (SD)	22.6 (10.6)	26.2 (12.1)	27.1 (12.8)	26.1 (13.0)	26.2 (13.3)	25.9 (12.6)	24.5 (17.7)	0.002
		p < 0.001	p = 0.189	p = 0.125	p = 0.779	p = 0.509	p = 0.260	
EMG_masseter in μV; mean (SD)	12.3 (10.5)	12.6 (9.4)	13.2 (9.9)	15.3 (10.5)	16.1 (13.0)	13.9 (9.4)	14.5 (13.8)	0.003
		p = 0.571	p = 0.185	p = 0.010	p = 0.201	p = 0.160	p = 0.570	
EMG_trapezius in μV; mean (SD)	44.4 (48.9)	44.0 (49.1)	45.7 (52.9)	45.5 (47.0)	44.1 (48.1)	42.2 (47.2)	35.3 (35.3)	0.002
		p = 0.816	p = 0.189	p = 0.923	p = 0.230	p = 0.280	p = 0.746	

1st and 2^nd^ challenge tasks with a self-determined speed and no social comparison. 1st and 2^nd^ complex tasks with computer determined speed and a comparison with no realistically high social ‘norm’. In bold represent the significant results. The overall results of the physiological parameters show F-values as listed below: Systolic blood pressure F(3.22, 444.74) = 39.76, p < 0.001, ƞ_p_^2^ = .23, ɛ = 1.00, diastolic blood pressure F(3.10, 427.42) = 47.66, p < 0.001, ƞ_p_^2^ = .26, ɛ = 1.00, heart rate F(3.74, 516) = 38.67, p < 0.001, ƞ_p_^2^ = .22, ɛ = 1.00. Breath depth F(2.65, 365.68) = 12.79, p < 0.001, ƞ_p_^2^ = .09, ɛ = .94, respiration rate F(2.92, 402.82) = 24.92, p < 0.001, ƞ_p_^2^ = .15, ɛ = .99. EMG_frontalis F(2.32, 320) = 6.07, p < 0.002, ƞ_p_^2^ = .04, ɛ = .78, EMG_masseter F(2.87, 392.55) = 4.88, p < 0.003, ƞ_p_^2^ = .03, ɛ = .77, EMG_trapezius F (3.32, 458.15) = 4.70, p < 0.002, ƞ_p_^2^ = .03, ɛ = .92.

**Table 4:** Psychological parameters: Repeated measures ANOVA.

psychological items	1^st^ challenge	1^st^ complex task	2^nd^ complex task	2^nd^ challenge	p (overall)
perceived body perception; mean (SD)	1.2 (1.1)	1.6 (1.2)	1.7 (1.2)	1.6 (1.2)	< 0.001
		p < 0.001	p = 0.555	p = 0.259	
the tasks are challenging me; mean (SD)	1.5 (1.1)	2.4 (1.1)	2.6 (1.2)	2.1 (1.2)	< 0.001
		p < 0.001	p = 0.006	p < 0.001	
the tasks are annoying; mean (SD)	0.8 (0.9)	1.1 (1.1)	1.0 (1.1)	0.8 (1.1)	0.002
		p = 0.064	p = 0.295	p = 0.008	
anger about my performance; mean (SD)	0.8 (1.0)	1.8 (1.5)	1.8 (1.4)	1.4 (1.3)	< 0.001
		p < 0.001	p = 0.737	p < 0.001	
anger about the tasks; mean (SD)	0.3 (0.6)	0.9 (1.1)	1.1 (1.2)	0.7 (1.1)	< 0.001
		p < 0.001	p = 0.128	p < 0.001	
fear of failure; mean (SD)	1.3 (1.2)	1.3 (1.2)	1.3 (1.4)	1.1 (1.3)	0.035
		p = 0.688	p = 0.778	p = 0.038	
being pleased with success; mean (SD)	1.9 (1.2)	0.6 (0.7)	0.7 (0.7)	1.5 (1.1)	< 0.001
		p < 0.001	p = 0.339	p < 0.001	

1^st^ and 2^nd^ challenge tasks with a self-determined speed and no comparison. 1^st^ and 2^nd^ complex tasks with a computer determined speed and a comparison with no realistically high social ‘norm’. In bold represent the significant results. The overall results of the psychological parameters show F-values as listed below: Perceived body perception F(2.62, 361.85) = 19.55, p < 0.001, ƞ_p_^2^ = .12, ɛ = .99, the tasks are challenging me F(2.54, 350.10) = 59.83, p < 0.001, ƞ_p_^2^ = .30, ɛ = 1.00, the tasks are annoying F(2.44, 336.48) = 5.64, p < 0.002, ƞ_p_^2^ = .04, ɛ = .91, anger about my performance F(2.38, 329.10) = 56.43, p < 0.001, ƞ_p_^2^ = .29, ɛ = 1.00, anger about the tasks F(2.33, 321.86) = 35.29, p < 0.001, ƞ_p_^2^ = .20, ɛ = 1.00, fear of failure F(2.54, 350.36) = 3.10, p < 0.035, ƞ_p_^2^ = .02, ɛ = .67, being pleased with success F(2.31, 318.18) = 90.88, p < 0.001, ƞ_p_^2^ = .40, ɛ = 1.00.

**Table 5:** Expectations and valence parameters: Repeated measures ANOVA.

	before 1^st^ challenge	after 1^st^ challenge	before 1^st^ complex task	before 2^nd^ complex task	after 2^nd^ complex task	before 2^nd^ challenge	after 2^nd^ challenge	p (overall)
**Ideal expectation; mean (SD)**	74.7 (19.3)	72.9 (20.3)	62.9 (17.9)	56.2 (20.6)	51.4 (22.9)	64.1 (22.7)	60.7 (22.8)	**< 0.001**
		p = 0.212	**p < 0.001**	**p < 0.001**	**p < 0.001**	**p < 0.001**	p = 0.327	
**Real expectation; mean (SD)**	63.7 (19.5)	66.3 (19.6)	53.7 (17.1)	41.3 (17.8)	36.8 (18.2)	55.1 (23.2)	50.3 (21.8)	**< 0.001**
		p = 0.620	**p < 0.001**	**p < 0.001**	**p < 0.001**	**p < 0.001**	p = 0.043	
**Relevance of success; mean (SD)**	70.0 (23.5)	63.3 (27.5)	58.8 (28.5)	55.1 (29.0)	53.6 (29.3)	56.5 (30.9)	55.9 (30.4)	**< 0.001**
		**p < 0.001**	**p = 0.004**	**p = 0.041**	p = 0.187	p = 0.537	p = 0.632	
**Relevance of failure, mean (SD)**	49.5 (29.2)	46.9 (31.5)	44.9 (30.3)	44.2 (31.6)	42.8 (32.5)	46.4 (33.6)	44.6 (32.3)	**0.004**
		p = 0.894	p = 0.105	p = 0.541	p = 0.190	p = 0.091	p = 0.173	

1^st^ and 2^nd^ challenge tasks with a self-determined speed with no comparison. 1^st^ and 2^nd^ complex tasks with a computer determined speed and a comparison with no realistically high social ‘norm’. In bold represent the significant results. The overall results of the expectations and valence parameters show F-values as listed below: Ideal expectation F(4.68, 645.31) = 60.31, p < 0.001, ƞ_p_^2^ = .30, ɛ = 1.00, real expectation F(4.25, 586.10) = 96.36, p < 0.001, ƞ_p_^2^ = .41, ɛ = 1.00, relevance of success F(4, 550.78) = 31.76, p < 0.001, ƞ_p_^2^ = .19, ɛ = 1.00, relevance of failure F(3.89, 467.68) = 4.25, p < 0.004, ƞ_p_^2^ = .03, ɛ = .89.

For all PPST periods (the varied conditions), means and standard deviations for the different variables (physiological and psychological parameters) and the p-values from the ANOVAs (indicated with bent arrows) are presented in Tables 2, 3, 4 and 5.

### Results of the mixed repeated-measures ANOVA

The overall results of the mixed repeated-measures ANOVA showed no significant differences between the total group of participants and the group of participants with either F30, F40 or F50 diagnoses. But we investigated significant within—subject factors. The following Figs (2–11) show the significant differences. They are marked with an asterisk.

**Fig 2:**
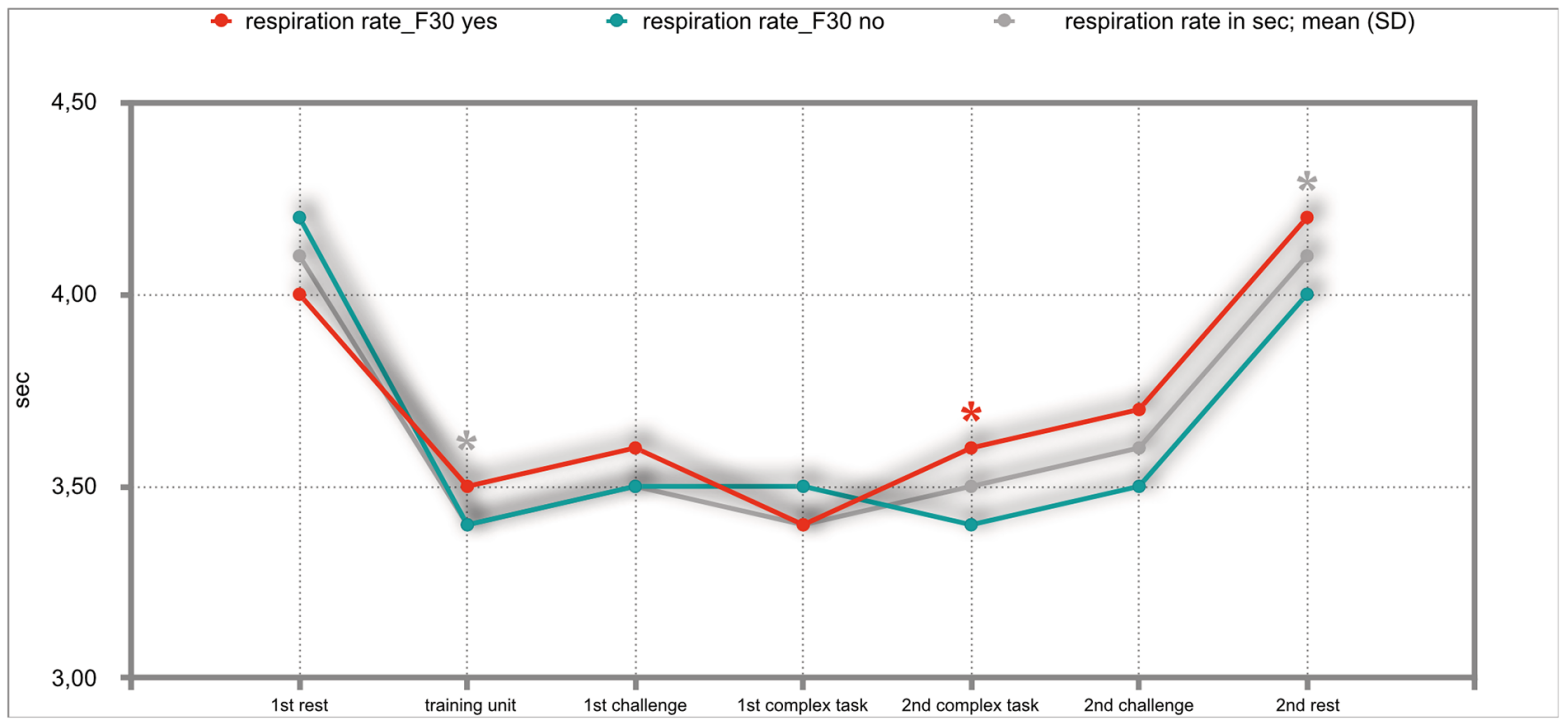
Respiration rate (total participants; F30-yes; F30-no). Challenge 1^st^ and 2^nd^: tasks with self-determined speed without a social comparison. Complex 1^st^ and 2^nd^ tasks with the computer-determined speed provided a comparison to an unrealistically high social ‘norm’.

**Fig 3:**
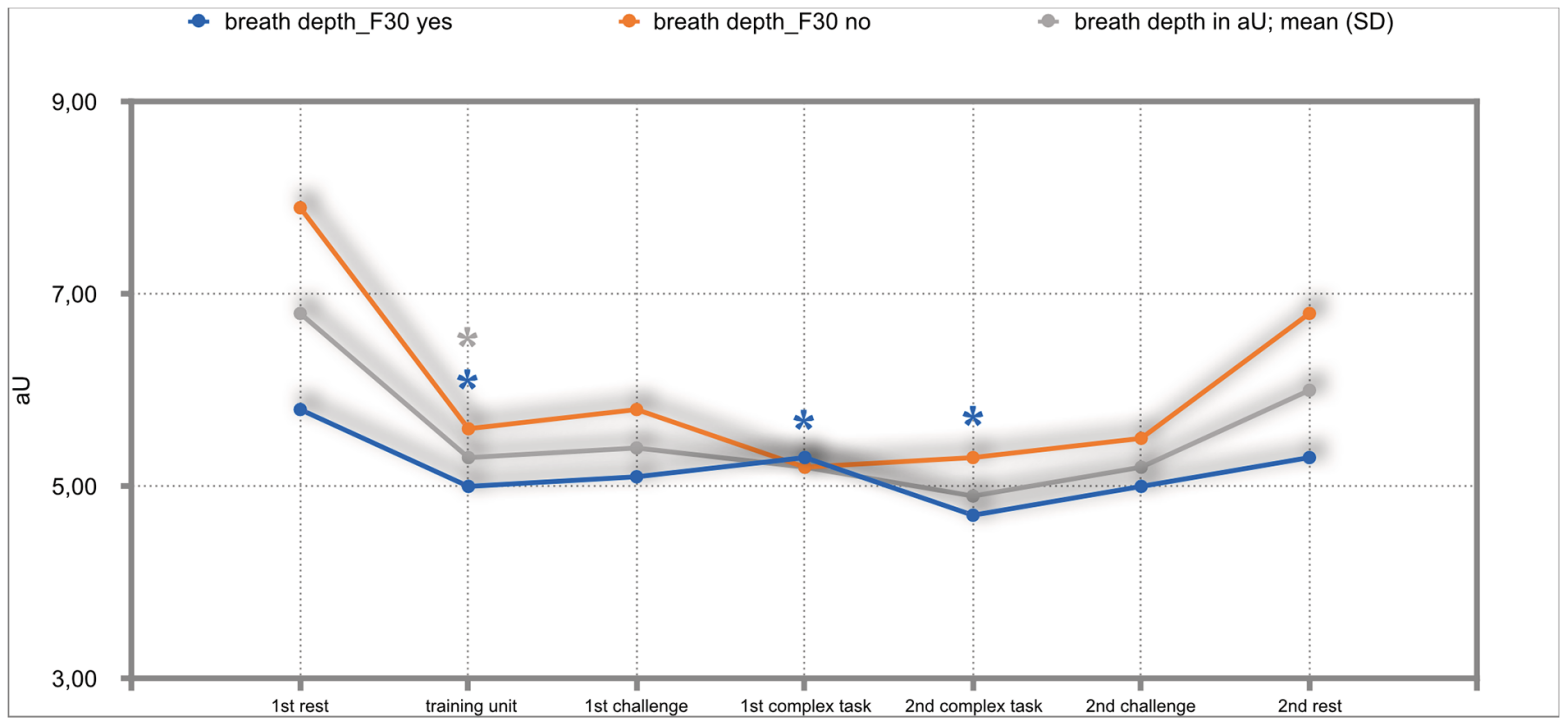
Breath-depth (total participants; F30-yes; F30-no). Challenge 1^st^ and 2^nd^: tasks with self-determined speed without a social comparison. Complex 1^st^ and 2^nd^ tasks with the computer-determined speed provided a comparison to an unrealistically high social ‘norm’.

**Fig 4:**
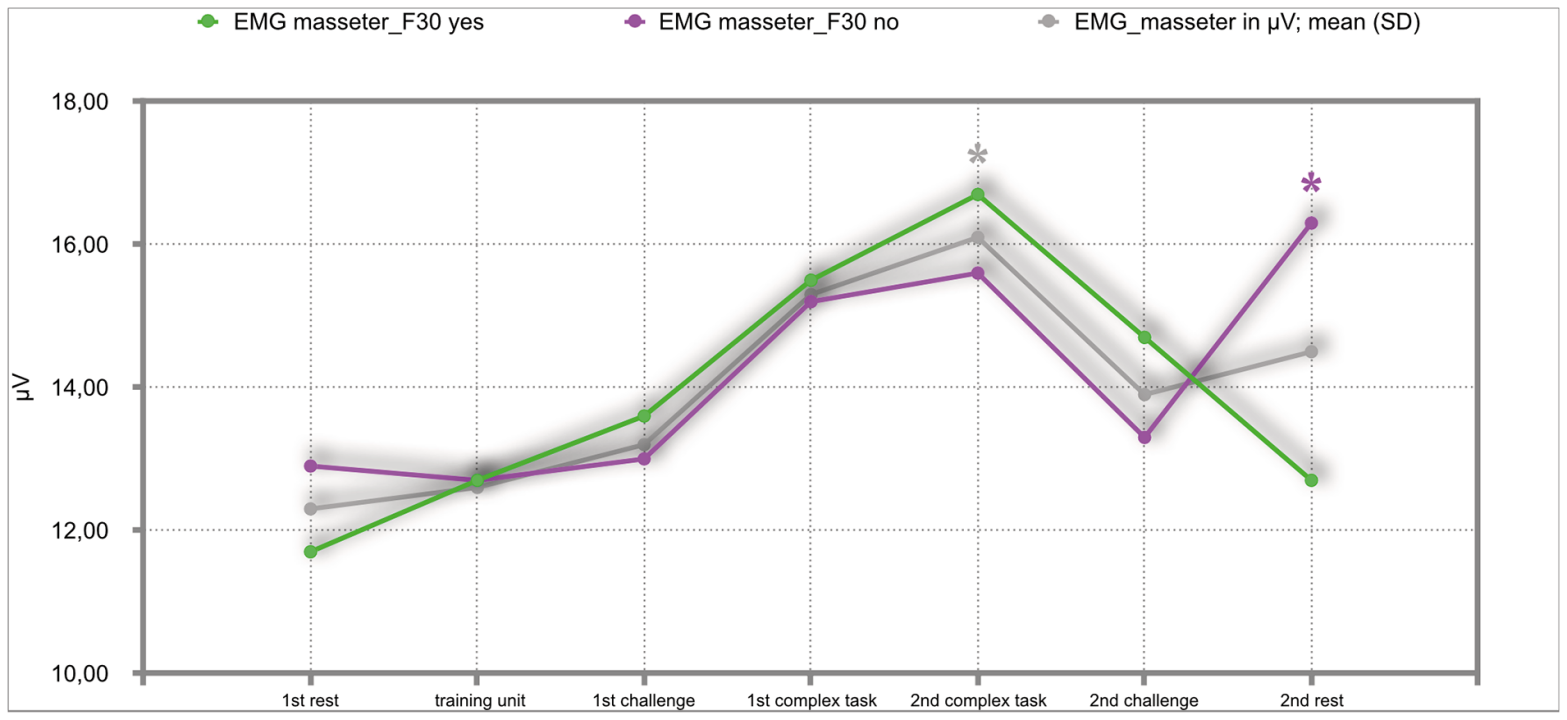
EMG-masseter (total participants, F30-yes; F30-no). Challenge 1^st^ and 2^nd^: tasks with self-determined speed without a social comparison. Complex 1^st^ and 2^nd^ tasks with the computer-determined speed provided a comparison to an unrealistically high social ‘norm’.

**Fig 5:**
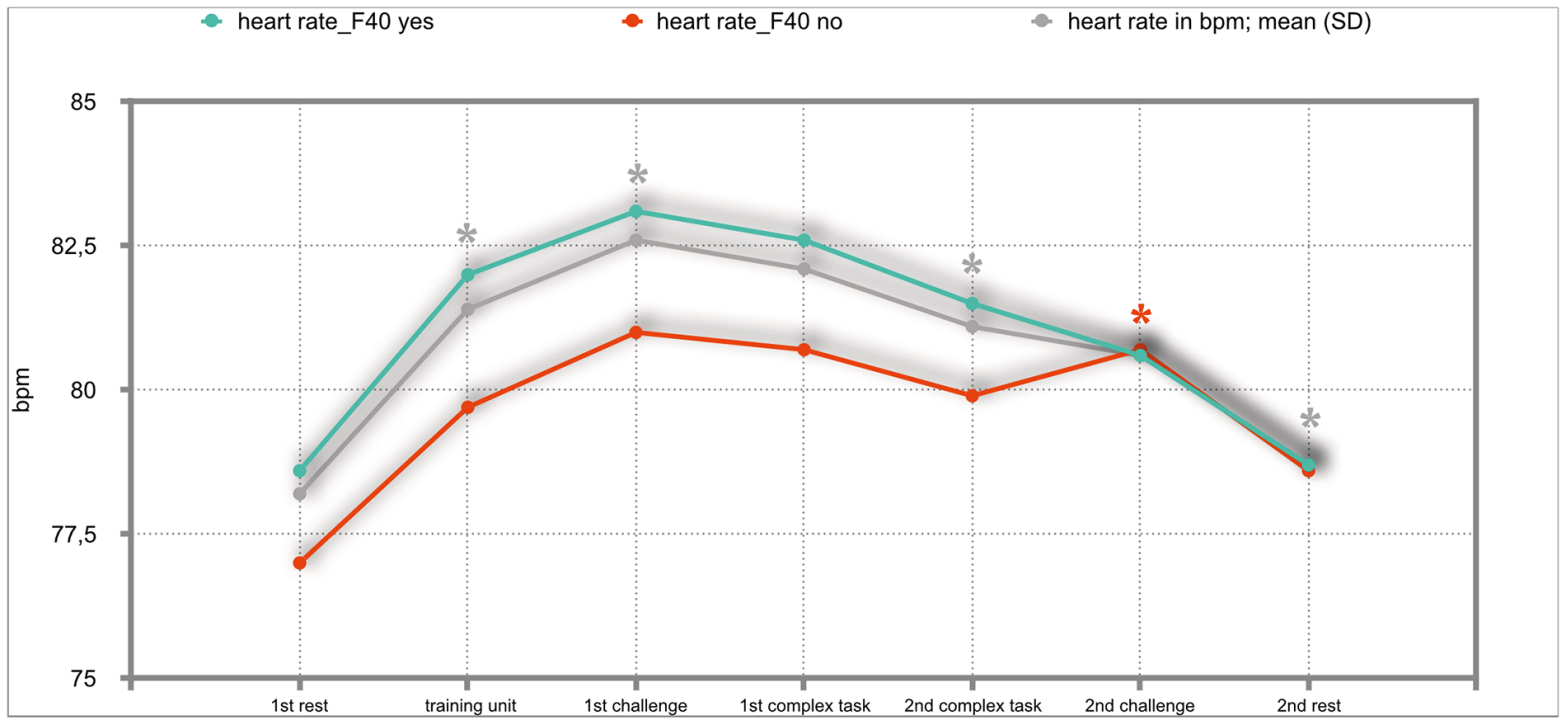
Heart-rate (total participants, F40-yes; F40-no). Challenge 1^st^ and 2^nd^: tasks with self-determined speed without a social comparison. Complex 1^st^ and 2^nd^ tasks with the computer-determined speed provided a comparison to an unrealistically high social ‘norm’.

**Fig 6:**
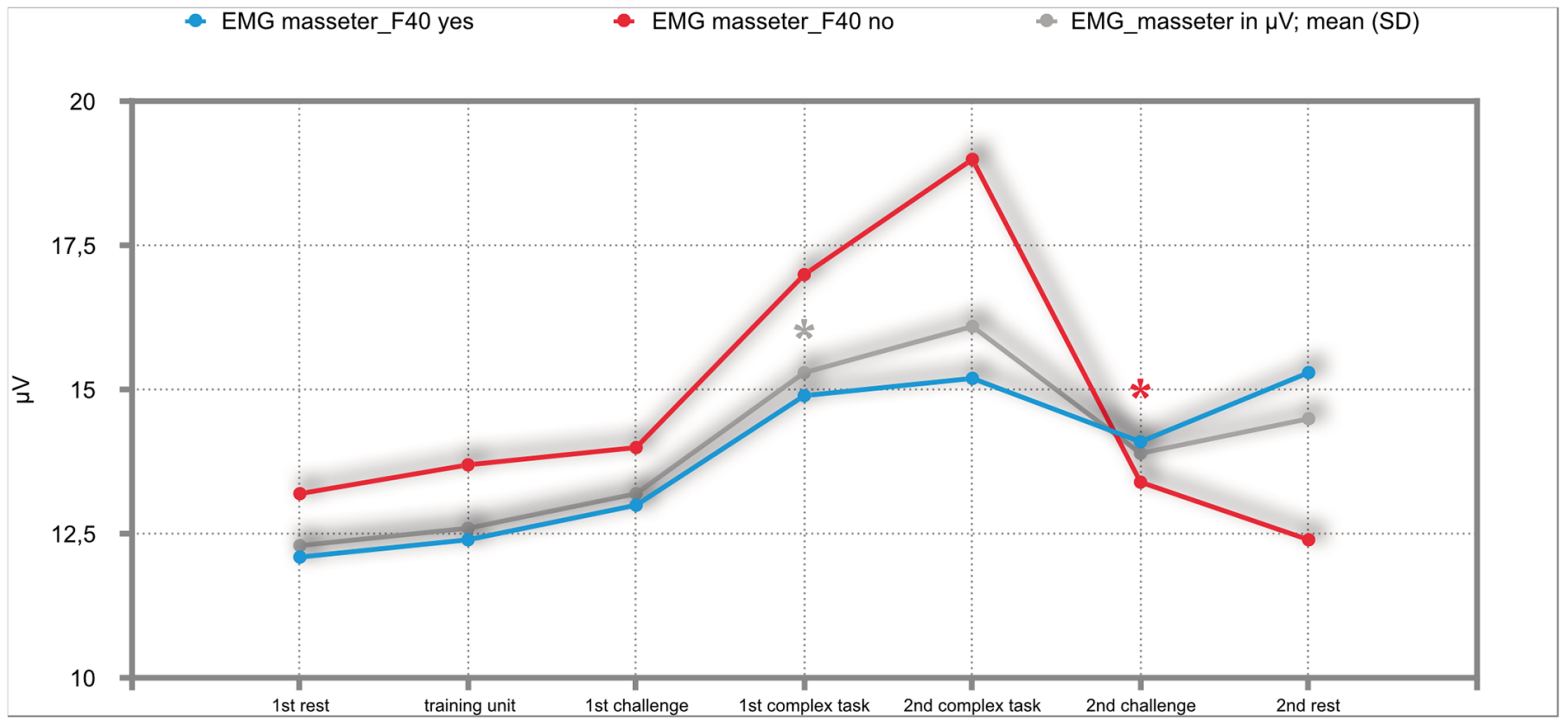
EMG-masseter (total participants; F40-yes; F40-no). Challenge 1^st^ and 2^nd^: tasks with self-determined speed without a social comparison. Complex 1^st^ and 2^nd^ tasks with the computer-determined speed provided a comparison to an unrealistically high social ‘norm’.

**Fig 7:**
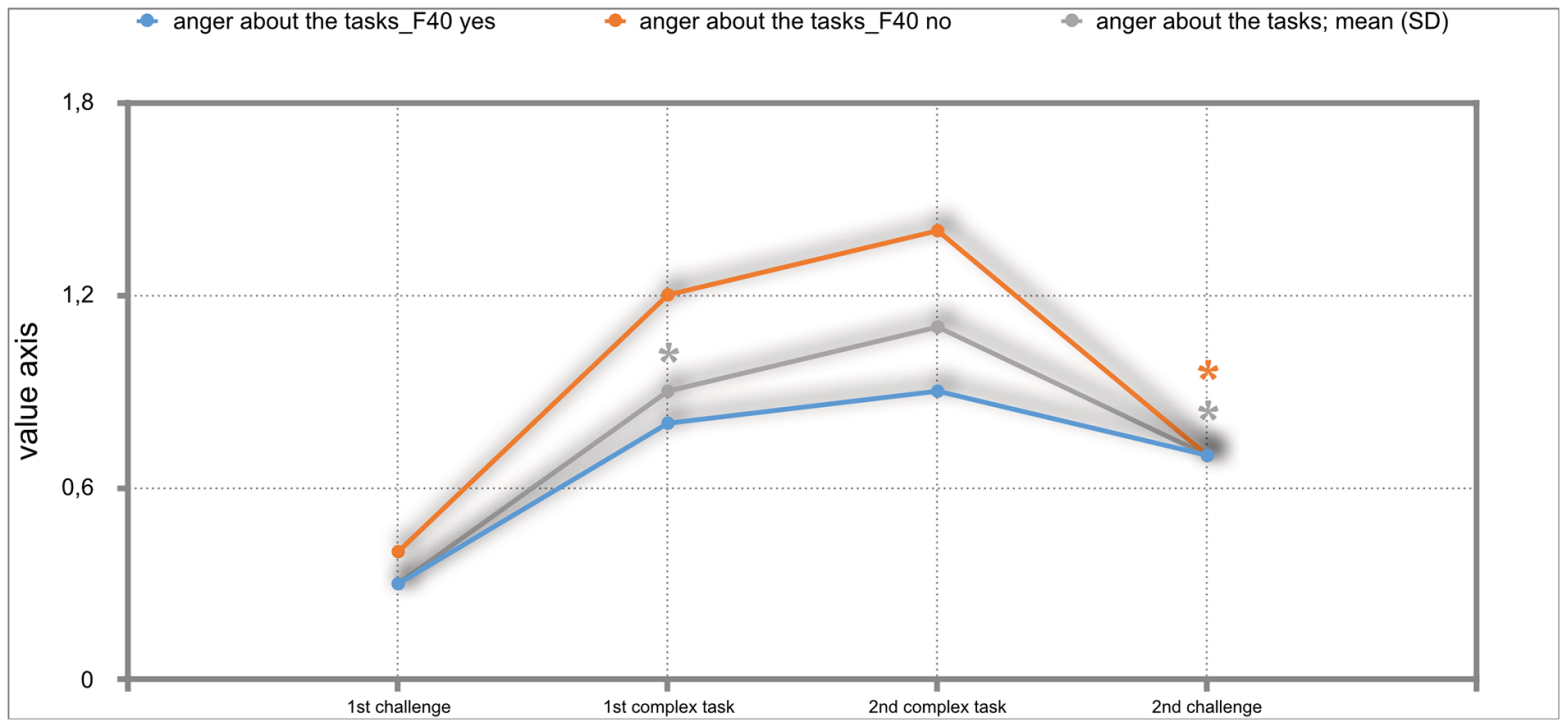
Anger about the tasks (total participants; F40-yes; F40-no). Challenge 1^st^ and 2^nd^: tasks with self-determined speed without a social comparison. Complex 1^st^ and 2^nd^ tasks with the computer-determined speed provided a comparison to an unrealistically high social ‘norm’.

**Fig 8:**
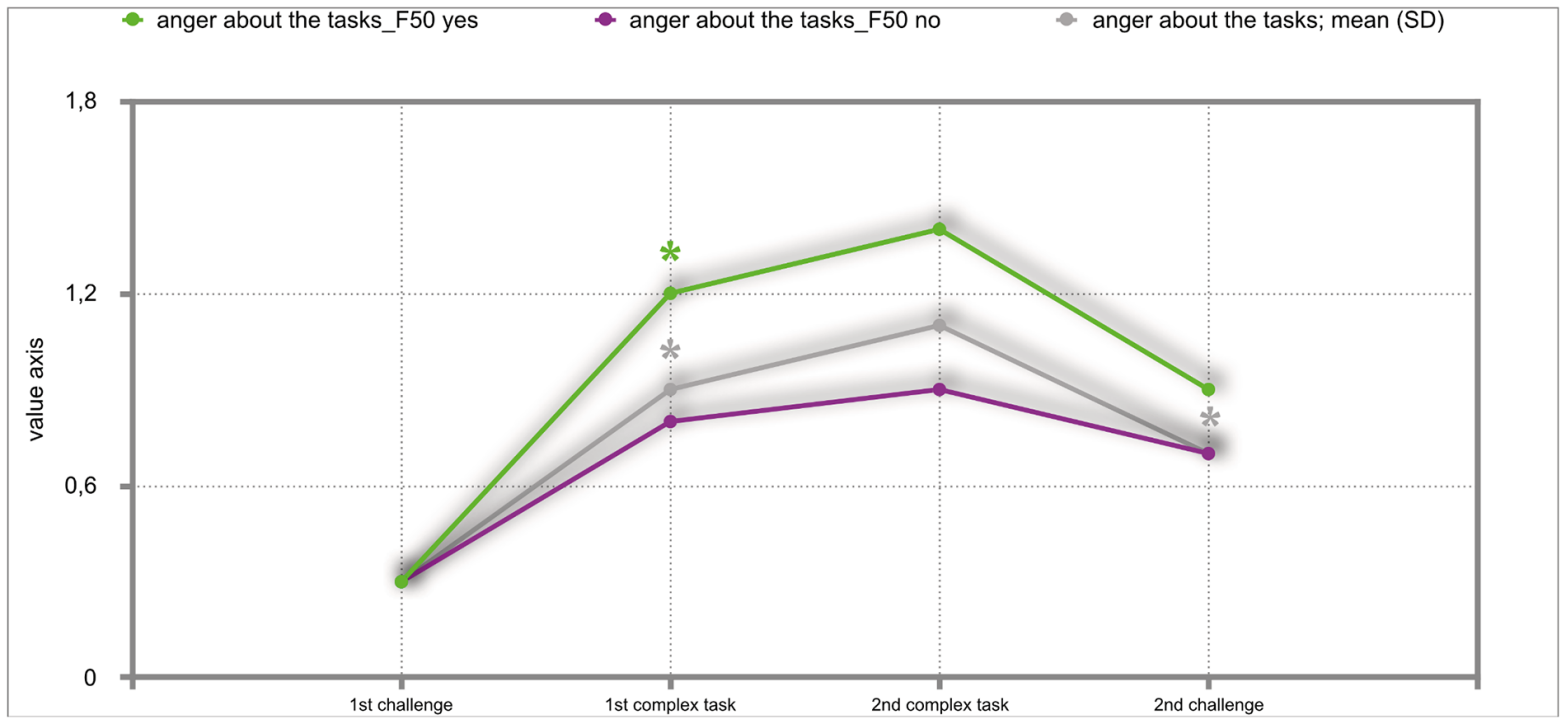
Anger about the tasks (total participants; F50-yes; F50-no). Challenge 1^st^ and 2^nd^: tasks with self-determined speed without a social comparison. Complex 1^st^ and 2^nd^ tasks with the computer-determined speed provided a comparison to an unrealistically high social ‘norm’.

**Fig 9:**
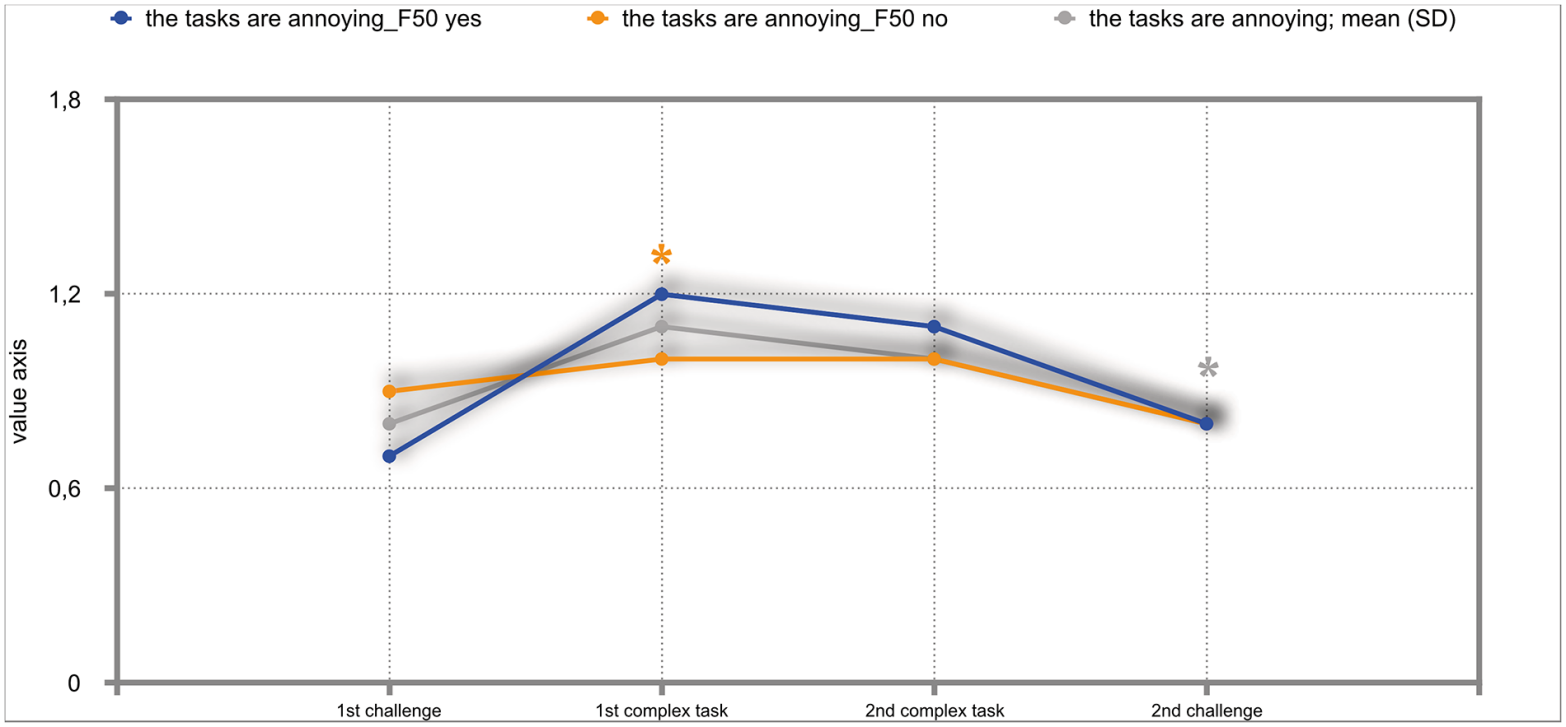
The tasks are annoying (total participants; F50-yes; F50-no). Challenge 1^st^ and 2^nd^: tasks with self-determined speed without a social comparison. Complex 1^st^ and 2^nd^ tasks with the computer-determined speed provided a comparison to an unrealistically high social ‘norm’.

**Fig 10:**
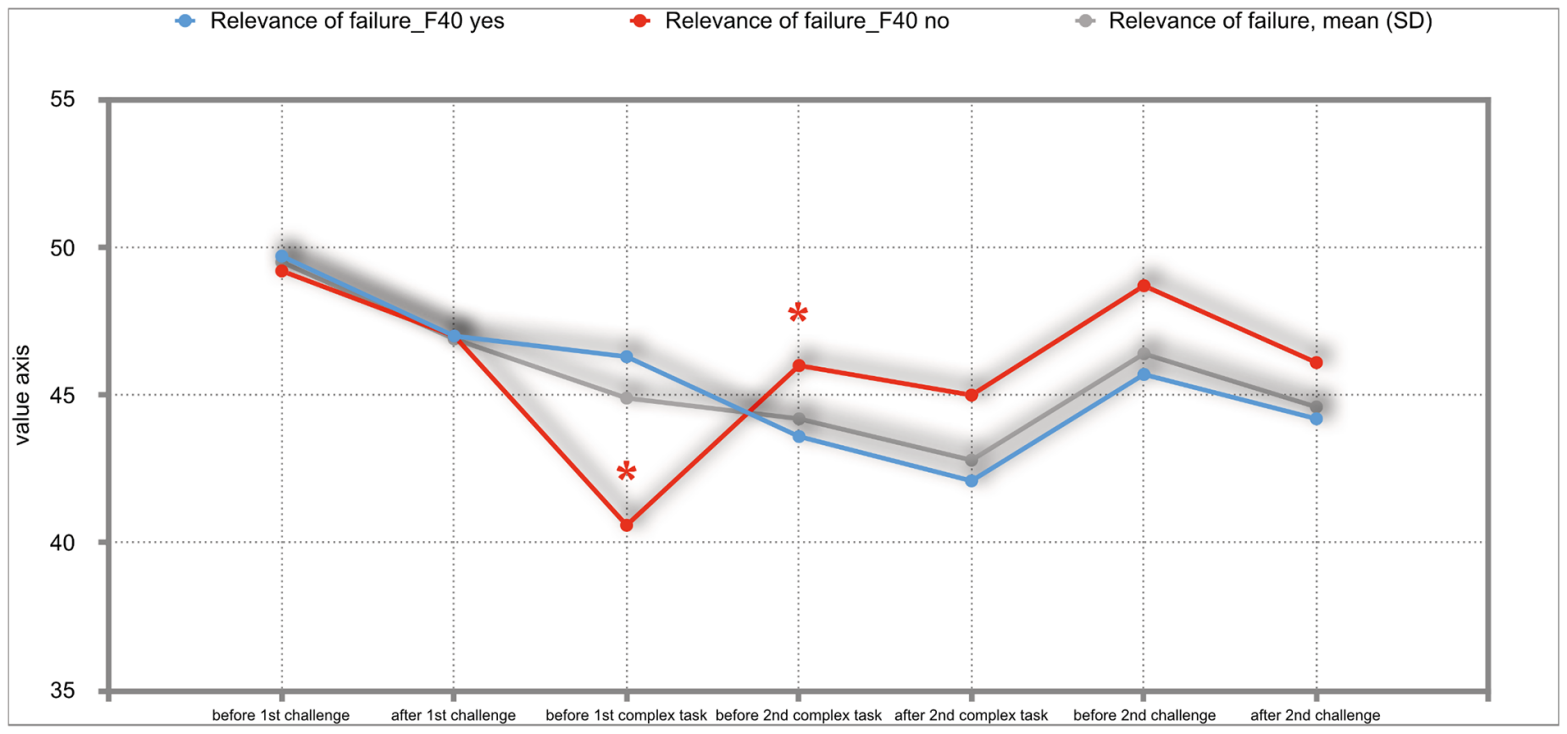
Relevance of failure (total participants; F40-yes; F40-no). Challenge 1^st^ and 2^nd^: tasks with self-determined speed without a social comparison. Complex 1^st^ and 2^nd^ tasks with the computer-determined speed provided a comparison to an unrealistically high social ‘norm’.

**Fig 11:**
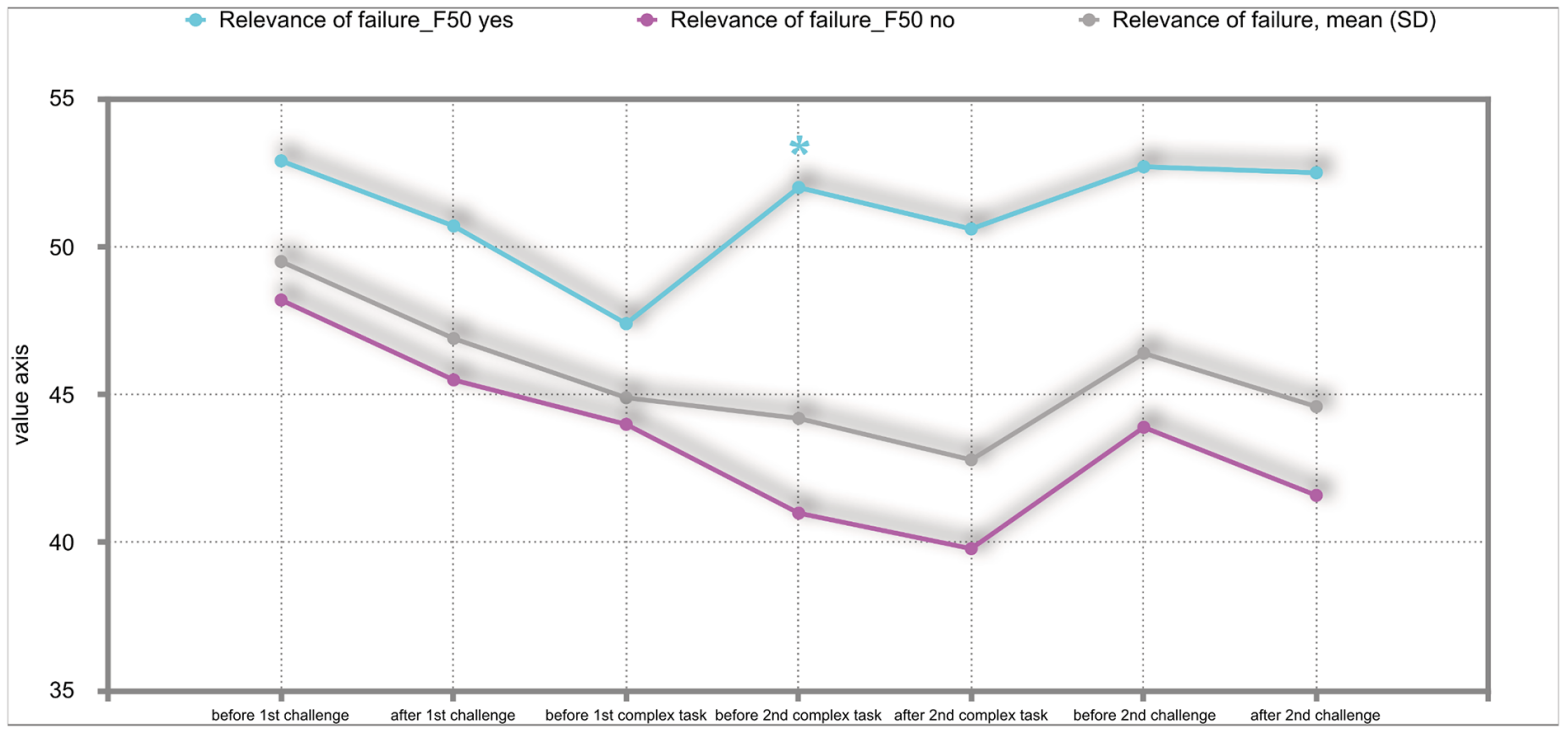
Relevance of failure (total participants; F50-yes; F50-no). Challenge 1^st^ and 2^nd^: tasks with self-determined speed without a social comparison. Complex 1^st^ and 2^nd^ tasks with the computer-determined speed provided a comparison to an unrealistically high social ‘norm’.

### Results summary

In this sample, the PPST showed the most prominent physiological reactions in the cardiovascular system (blood pressure and heart rate) throughout the examination (Table 3). Further, we found that the different testing conditions provoked adjustments at the different psychological levels (cognitive: performance, expectations, and valence) (Tables 2 and 5). Hence, comparing the results for the challenge periods at the beginning and end of the test shows that in the 2^nd^ challenge period (after both complex task periods), patients’ estimations of performance, ideal and real expectations significantly decreased. Further, to appraise the different testing conditions, we assume that the PPST evokes a stress experience. According to Lazarus [22], the individual´s appraisal of the stress-provoking situation is essential. The uncontrollable complex task testing periods negatively influence patients’ self-estimations of their performance; thus, the valences and expectations decline significantly. For the physiological parameters, the statistical analysis shows significantly increased activity for the cardiovascular system reactions (systolic blood pressure, heart rate) in the complex task periods, whereas for the other physiological variables (EMG, breath depth, respiration rate, diastolic blood pressure) differences in the complex task periods are not significant.

## Case example

**Fig 12:**
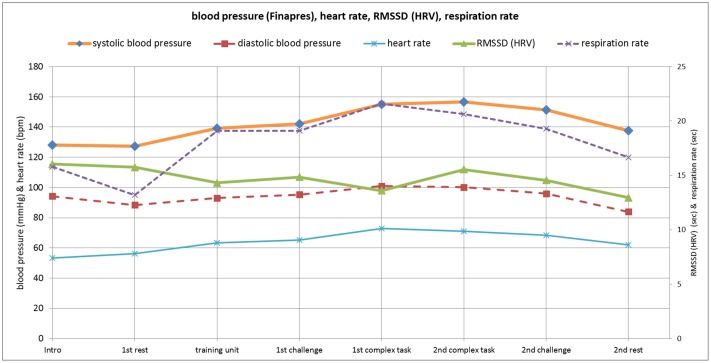
Patient M.M..; 19 y.; ICD 10: F50.0: Blood pressure, heart rate, respiration rate and RMSSD. Challenge 1^st^ and 2^nd^: tasks with self-determined speed without a social comparison. Complex 1^st^ and 2^nd^ tasks with the computer-determined speed provided a comparison to an unrealistically high social ‘norm’. Blood pressure values by Finapres leads to falsely high values—since gauging is missing—and are suited only for depicting an uninterrupted course. Therefore some measurements of blood pressure via the riva rocci method are taken additionally for clinical comparison: 1^st^ period of rest– 88/62 mmHg, 1^st^ complex task– 103/65 mmHg, 2^nd^ complex task– 99/60 mmHg, 2^nd^ period of rest– 89/56 mmHg.

**Fig 13:**
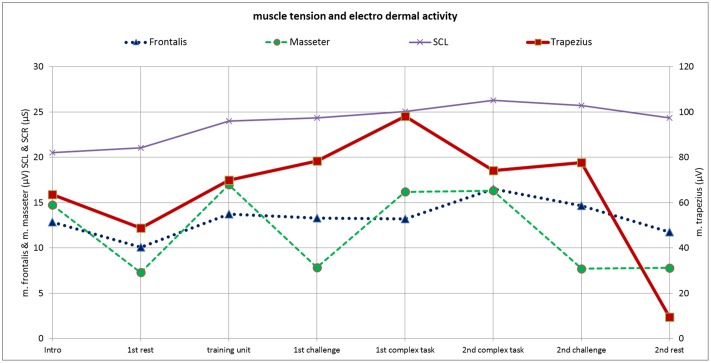
Patient M.M.; 19 y.; ICD 10: F50.0: Muscle tension—m. frontalis, m. masseter, m. trapezius, and electro dermal activity (SCL).

We show the graphs of a 19-year-old female student who was diagnosed with anorexia nervosa—ICD 10: F50.0—(BMI 14 kg/m^2^) that lasted for six months. The patient reported that she is under permanent pressure to perform her studies but is not able to achieve good results due to her eating disorder as well as her physiological and psychological condition. Her stress reactions and patterns show noticeable psychophysiological patterns (Tables 6 & 7 Figs 12 & 13), which are obscured when the data are shown at the aggregate level because each patient reacts differently. The patient’s treatment attempted to ameliorate her health complaints with psychotherapy in single and group setting, and creative therapy (i.e., music therapy, art therapy, relaxation techniques, and body perception centered therapy). Inpatient treatment lasted for approximately three months.

**Table 6:** Performance data from the case-report.

	total number presented	correct solutions	correct solutions	patients’ estimation	time per matrix	matrices	correct solutions
test periods	n.	n.	% of total number	%	sec	per min	per min
*training unit*	16	11	69		7.6	7.9	5.4
*1*^*st*^ *challenge*	14	12	86	50	9.0	6.7	5.7
*1*^*st*^ *complex task*	31	12	39	10	3.9	15.3	5.9
*2*^*nd*^ *complex task*	31	12	39	12	3.9	15.4	6.0
*2*^*nd*^ *challenge*	14	12	86	50	9.1	6.6	5.6

**Table 7:** Expectation and valence data from the case report.

	ideal and real expectation answers 0–100%		relevance of success or failure answers 0–100%	
test periods	ideal	real	success	failure
*before 1*^*st*^ *challenge*	99	27	99	50
*after 1*^*st*^ *challenge*	95	30	95	95
*before 1*^*st*^ *complex task*	80	20	100	29
*before 2*^*nd*^ *complex task*	99	10	99	50
*after 2*^*nd*^ *complex task*	95	11	98	50
*before 2*^*nd*^ *challenge*	100	50	100	95
*after 2*^*nd*^ *challenge*	90	40	99	50

Due to the bad physical condition of the patient, we expected a delayed adaption for almost all of the physiological variables. Concerning the different dimensions of the psychological levels, we expected differences between her real performance and her self-estimation of performance, as well as a large discrepancy between the real and ideal expectations, because it is well known that patients who suffer from anorexia nervosa are characterized by a sense of ineffectiveness and perfectionism [23]. Additionally, we expected that success and failure would be almost equally important for the patient.

We assumed that there would be differences between the physiological reactions and her appraisal of her own body perception, because of the body image failure, which is a criterion of anorexia nervosa. Because of the need of self-control, we expected that the patient would report a higher fear of failure in the complex task periods than in the challenge periods.

### Measuring performance and the ability to concentrate

The patient processed 14 matrices in the 1^st^ challenge period (average 12.5 +/- 3.92), with the best performance found at 86% correct answers. According to the test construction, performance in both complex task periods is lower than in both challenge periods. In the 2^nd^ challenge phase, the patient processed again 14 matrices with 12 correct solutions (86%), which was the exact same performance as in the 1^st^ challenge period. This result indicates that this patient did not experience training effects or improvements in concentration during the test. In all test periods the patient estimates her performance lower than she achieved.

### Physiological parameters

The continuously measured *systolic and diastolic blood pressure* values show a parallel course, with increases from the 1^st^ rest period until the 2^nd^ complex task. From the 2^nd^ complex task period until the 2^nd^ rest period, the levels decrease.

The *heart rate* shows almost the same course as the blood pressure, with an earlier climax in the 1^st^ complex task phase. The *RMSSD (HRV)* only had slight fluctuations throughout the examination, with a climax in the 1^st^ rest period and the 2^nd^ complex task period. *Respiration rate* had the highest value in the 1^st^ complex task period. The continuously measured systolic and diastolic blood pressure values are high for a woman of her age who has an anorexia nervosa diagnosis with a BMI of 14 kg/m^2^. These values are higher compared to the values of the other patients of our study (Table 3).

The *m*. *trapezius* activity increases from the 1^st^ rest period to the 1^st^ complex task period and then decreases until the 2^nd^ complex task period, holds this level in the 2^nd^ challenge phase, and then steeply declines in the 2^nd^ rest period to a lower level than in the 1^st^ rest phase. This may be seen as a ‘delayed adaptive’ curve: after activation up to a climax, the course does not show a continuously progressive decline but instead creates a ‘shoulder’. In comparison, an ‘early adaptive’ course occurs when a continuously progressive decline follows activation, which we could find for respiration rate, heart rate, and systolic and diastolic blood pressure (see above).

The *m*. *frontalis* had the highest activity in the 2^nd^ complex task period, after which the activity continuously declines until the 2^nd^ rest period. Waterink & van Boxtel [24] postulated that the facial EMG uninterruptedly increases when the participant’s performance is stable. The m. frontalis’ (Fig 13) course shows an uninterrupted increase until the 2^nd^ complex task period, when the patient’s performance is stable (Table 6).

The *m*. *masseter’s* course is marked by alternating steep increases and declines throughout the test.

*Skin conductance level* (SCL) continuously increases until the 2^nd^ complex task period with only slight decrease thereafter, representing a ‘delayed adaptive’ curve.

### Emotional experiencing

**Fig 14:**
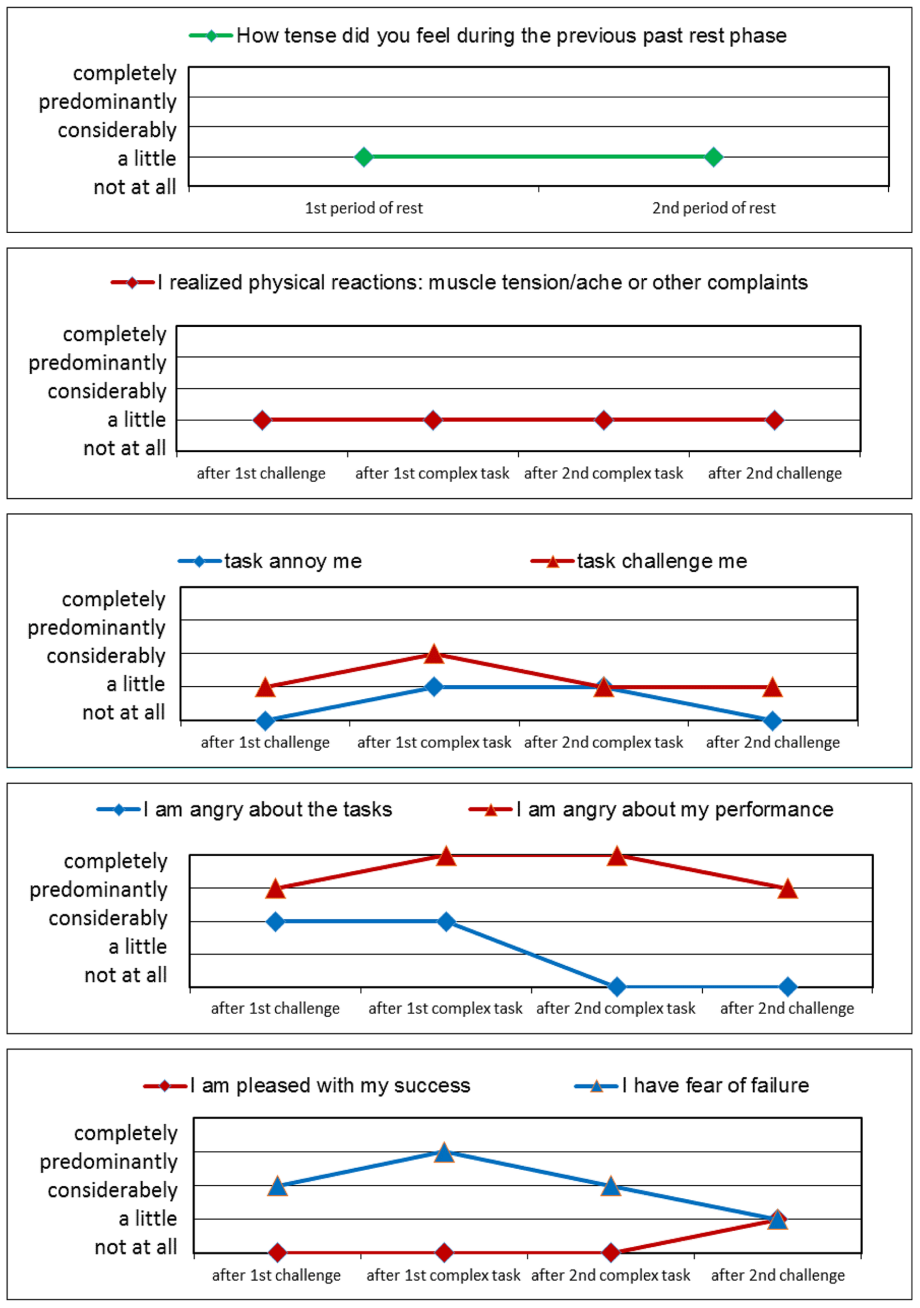
Patient M.M.; 19 y.; ICD 10: F50.0: Psychological data graphs.

The patient reports scarce *physical reactions*. (Fig 14) She stated ‘a little’ tenseness after the rest periods. Throughout the test, the patient identified ‘a little’ muscle tension or other body-related discomforts.

For the ‘*tasks challenge me*’ question, she reported ‘considerably’ after the 1^st^ complex task period and ‘a little’ for all other periods. She did not report *‘annoyance’* except for the complex task periods.

*Anger about her performance* was reflected by reporting the maximum values for ‘predominantly and completely,’ whereas *anger related to the tasks* was rated lower and reported as ‘not at all’ in the second half of the test. She reported the minimum values (‘not at all, a little’) for *being pleased with her success* and reported a considerable *fear of failure*.

The patient reports *maximal ideal expectations* throughout the test. Contrasting her *real expectations* show low values. When the computer algorithm was activated and the instructions indicated that her peer group performed at approximately 60% correct, her ideal expectation remains high level (80% or more). In contrast, her real expectation declines to 20% and 10%. Therefore, her ideal and real expectations sharply diverge across testing periods. The *importance of success* is rated with the maximum values throughout the test. The values for the *importance of failure* fluctuate heavily, with the highest values (95%) after the 1^st^ and before the 2^nd^ challenge phases and the lowest value (29%) before the 1^st^ complex task.

### Case example summary

We found a ‘delayed adaption’ in activation of the m. trapezius and m. masseter, whereas the other physiological variables showed an ‘early adaptive’ course (Figs 12 and 13). Thus, we assume that the patient perceives her own physiological body reactions (Fig 14) differently from the physiological measures (Figs 12 and 13). The body perception impairment reflects a body image failure. Her performance self-estimation and (real) performance values show differences. The patient reported high levels for fear of failure. We view the psychological variables as person-related—internal factors, including ‘I am angry about my performance,’ ‘tasks challenge me,’ ‘I am pleased with my success’ and ‘I have a fear of failure’, compared with external factors, such as ‘tasks annoy me’ and ‘I am angry about the tasks’. This patient scores higher on internal factors, which may indicate a need for person-related coping strategies to better adjust to the varied stress periods. The discrepancy between real- and ideal expectations is large and success is important to the patient (high importance of success). In contrast the importance failure looks down-regulated, which further reflects her internal person- related coping strategies. Consequently, we stressed the importance of relaxation techniques and encouraged the patient to practice these techniques and gain experience in body centered creative therapy (i.e., music & art therapy).

## Discussion

The PPST is supposed to reflect psychophysiological reactions in an artificial situation where challenging tasks must be performed under time pressure, which may be comparable with daily life stress, e.g., at work. There is a large body of literature that postulates the problem of transferability of results of experimental studies and observations of people’s behavior in real life [25–28]. Stress examinations in laboratory situations are unavoidably reductionistic; therefore, it is complicated to transfer the results into the daily lives of patients. Most patients are involved in daily life with different stressors simultaneously (i.e., psychosocial stressors)–the so-called daily hassles [29]. However, we must consider that inpatient treatment and waiting for the results of the physiological tests may induce comparable stress reactions. Hence, to be in inpatient treatment has an influence on physiological and psychological systems. Patients with distress experiences in daily life are often exhausted, and their treatment should tend to recover their impairments. The reading, analysis and interpretation of the PPST results are supposed to serve or to promote patients’ introspection and self-reflection regarding their individual bio-psycho-social conditions. The encouragement of introspection and self-reflection can be an advantage concerning the further ambulatory treatment. That is, the better the patient knows his/her patterns of stress reaction, the better he/she applies discussed coping strategies in his/her daily life. Our results show that the PPST allows psychophysiological stress reactions to be measured in a laboratory situation, even though our results cannot represent general population. Previous studies of the PPST—using our previous protocol that had four additional challenge periods—have shown that there are comprehensive influences concerning the reactions and stress patterns of special diagnostic groups such as depression or back pain [30–33], we are now focused on reproducing the results with the new modified protocol.

We could demonstrate the PPST showed stress-related changes in several dimensions as results at the group level. However, PPST’s main advantage is its qualities at the individual level because it identifies stress patterns and has the potential to give the patients a better understanding of their psychophysiological condition and discussion of outpatient treatment options. Beyond the horizon of psychophysiological studies, the PPST may be helpful in approaches of so called mindfulness. Because the PPST provokes psychophysiological stress reactions, as shown here, the PPST can be used as a standardized instrument in stress diagnostics, and its need for only one examiner is beneficial concerning the personal resources in clinical departments and practice.

### Limitations

We adapted the protocol into seven periods because the examination was extremely exhausting for the patients and because the investigators (psychologist, medical doctors) have less time resources for such a long experimental session. This modification could have an impact, or limitation on our group results because the timeframe may be too short to provoke stronger stress reactions and the following psychophysiological adaptation process or delayed adaptations are not comprehensively demonstrated in the group statistic. Due to the clinical routine, the participants performed the test not exactly at the same time—two-thirds of the patients performed the test in the afternoon and one-third in the morning. This may be a contributing factor in terms of our results. Another limitation of our study may be that we have no healthy group sample for comparison of our results, this is concerning in terms of the validity of the PPST. Our results illustrate patients with a likely impaired psychophysiological condition. The participation in the PPST was prescribed by our medical doctors and psychologist for diagnosing stress reactions, we had no inclusion or exclusion criteria to control the variance of our participants, and this might have also an influence on our statistical analysis.

### Outlook

For the next future, we intend to combine the PPST as a performance-centered test with an evaluation of primarily emotional challenge / stress in a creative therapeutical setting, such as music therapy, to elicit / provoke different stimuli-specific reactions in patients. So, the patients undergo a wider range of stress experience and the analysis of stress patterns and stress reactions are probably more comprehensive. Since prior research indicated that psychophysiological stress reactions and patterns are individual, we will examine in the next step whether individuals from a healthy control group and special disease groups differently react to the test.

## Supporting information

S1 File Background data which was used for the analysis is publicly available. Please see corresponding file.
(SAV)
